# The influence of polystyrene nanoparticles on the fractal kinetics of lactate dehydrogenase

**DOI:** 10.1016/j.bbrep.2020.100793

**Published:** 2020-08-02

**Authors:** J. Auclair, F. Gagné

**Affiliations:** Aquatic Contaminant Research Division, Environment and Climate Change Canada, 105 McGill, Montreal, Québec, H2Y 2E7, Canada

**Keywords:** Lactate dehydrogenase, Fractal kinetics, Fractal dimension, Nanoplastics, Biophysical effects

## Abstract

Plastics are ubiquitous in the aquatic environment and their degradation of fragments down to the nanoscale level have raised concerns given their ability to pervade cells. The accumulation of nanoparticles could lead to molecular crowding which can alter the normal functioning of enzymes. The purpose of this study was to examine the influence of polystyrene nanoparticles (NPs) on the fractal kinetics of the lactate dehydrogenase reaction: pyruvate + NADH ↔ lactate + NAD^+^. The influence of NPs on LDH activity was examined first in vitro to highlight specific effects and secondly in mussels exposed to NPs *in vivo* for 24h at 15 °C. The reaction rates of LDH were determined with increasing concentrations of pyruvate to reach saturation at circa 1 mM pyruvate. The addition of F-actin, a known binding template for LDH, revealed a characteristic change in reaction rates associated with fractal organization. The addition of 50 and 100 nm transparent NPs also produced these changes. The fractal dimension was determined and revealed that both F-actin and NPs reduced the fractal dimension of the LDH reaction. The addition of viscosity sensor probe in the reaction media revealed viscosity waves during the reaction at low substrate concentrations thought to be associated to synchronized switching between the relaxed and tensed states of LDH. The amplitude and the frequency of viscosity waves were increased by both NPs and F-actin which were associated with increased reaction rates. In mussels exposed to NPs, the isolation of digestive gland subcellular fraction revealed that LDH activity was significantly influenced by the fractal dimension of the LDH reaction where a loss of affinity (high fractal K_M_) was detected in mussels exposed to the high concentrations of NPs. It is concluded that polystyrene NPs could change the biophysical properties of the cytoplasm such as the fractal organization of the intracellular environment during the LDH reaction.

## Highlights

• Polystyrene nanoparticles introduce crowding effects.
• The fractal kinetic of Lactate dehydrogenase in influenced by the plastics nanoparticles.
• These changes were also observed in mussels exposed to plastic nanoparticles.

## 1 Introduction

The increasing and continuous release of plastic materials in our water bodies represents a major contamination problem in the 21st century. Over 8 million tons of plastic materials are released in oceans per year and are broken down into smaller and smaller fragments over time [19]. Microplastics are fragments where one dimension is in 5 mm to 1 um range scale while nanoplastics (NPs) are fragments <1 um, usually between 100-1 nm in size [16,23]. In contrast with microplastics, NPs have the ability to permeate in cells and reach the cytoplasm. On the one hand, plastics are considered biologically and chemically inert materials i.e, producing no chemical reactions involved in toxicity such as production of reactive oxygen species and adduct formation to proteins and nucleic acids. On the other hand, the accumulation of NPs or other nanoparticles in the cytoplasm could produce crowding effects. Increased crowding or changes in the crowding space could disrupt the physical organisation of protein networks in cells. Indeed the toxicity of nanoparticles do not arise solely based on the chemical composition of the polymers but to other properties such as nanoparticle size/form, surface reactivity and vectorization of adsorbed chemicals [14]. The sheer physical presence of NPs in the already crowded environment of the cytoplasm could disrupt cellular processes including allosteric effects in multiple enzyme complexes. The effects of chemically inert NPs on the space organization in cells are largely understood at present time. A recent review examined the capacity of nanoparticles to induce protein fibrillation, which occurs in many degenerative disorders such as amyloid diseases [9]. It was shown that protein fibrillation and misfolding was a function of nanoparticle surface area (size) and surface charge. In another study, exposure to silver nanoparticles lead to increase levels in the protein chaperones-heat shock proteins 72 which suggests increased misfolding of proteins [21,22] and denaturation [13]. The contribution of crowding effects to protein denaturation were not determined.

Biochemical reactions involving enzymes have traditionally been analyzed using the mass-action approach of Michaelis-Menten kinetics from which enzyme activity are determined in ideal conditions, i.e. in dilute solution with saturating levels of substrates which is dominated by free Brownian motion [[2], [4]]. However, the intracellular environment is a crowded environment (estimated protein concentration between 100-500 mg/mL) where enzyme-catalyzed biochemical reactions occur in restricted space. This results in variations of biochemical reactions that do not follow the classical mass-action kinetics [24]. The cytoskeleton of the cytoplasm is organized as a maze-like similar to a random percolation (connected) fractal (Aon al, 1994). A fractal is broadly defined as pattern (a surface motif) that is scale invariant i.e., is repeated at different scales [20]. One of the consequences of a fractal organized cytoplasm is that enzyme reactions exhibits higher rates in fractal space at low substrate concentration and short reaction times than expected in classical Euclidian space. This organization enables cells to modulate metabolism at low substrate concentrations by altering the space domain by cytoskeleton spreading and rearrangement. Hence, fractal organization of a biochemical pathway allows to increase the flux during low levels of intermediate metabolites. Lactate dehydrogenase (LDH) is found in virtually all eukaryotic and prokaryotic cells and catalyzes the reversible reaction: pyruvate + NADH ↔ lactate + NAD^+^. The last step of anaerobic glycolysis is involved in the production of NADH in the absence of oxygen. LDH consists of tetramer (i.e., composed of 4 subunits) and exists in 2 conformational states: a relaxed and tensed state which is involved in catalysis. LDH is often used as a marker of anoxic conditions which can arise from the clogging effect of plastic compounds or other fine particles [27]. The enzyme is mainly found in the cytosol and forms dissipative structures (temporary enzymes-protein complex) with pyruvate kinase and F-actin for enhanced reaction rates in crowded intracellular environments [6,8]. Hence, its relevance to study the effects of the fractal properties of the intracellular milieu based on fractal kinetics.

The purpose of this study was therefore to examine the biophysical effect of neutral polystyrene NPs on LDH activity in vitro and in mussels exposed to plastic NPs. The null hypothesis is that NPs have no influence on the fractal properties of space involved in the LDH reaction. The observed effects in vitro will also be compared in freshwater mussels exposed to NPs in water. The biophysical effects of NPs on the fractal kinetics of the LDH reaction are identified in exposed mussels plastic NPs.

## 2 Materials and methods

### 2.1 In vitro LDH activity

Lactate dehydrogenase (LDH; rabbit muscle, type IX, lyophilized powder, 900 units/mg protein), pyruvate and reduced NADH were purchased from Sigma Chemical Company (Mississauga, ON, Canada). Polymerized F-actin were produced as follows: G-actine (500 ug/mL) was allowed to polymerize overnight at 4 °C in the following media: 100 mM KCl, 0.2 mM ATP, 2 mM MgCl_2_, 0.2 mM CaCl_2_ in 10 mM Hepes-NaOH, pH 7.5. The formation of F-actin was determined by absorbance at 450 nm. The activity in LDH were determined at 1 mM NADH and pyruvate at 25 °C in the assay buffer composed of 50 mM NaCl, 10 mM Hepes-NaOH, pH 7.5 with 0.1 μg/mL of commercial LDH. The activity was also measured with varying concentration in pyruvate (0.1 to 1 mM) in the presence of 1 mM NADH to determine the fractal kinetic constants (K_M_ and V_Max_) as described below. The reaction was allowed to proceed for 30 min where NADH fluorescence (350 nm excitation and 450 nm emission) were measured each 30 s under flash mode for quick readings in dark 96-well microplates (Synergy-4, Biotek Instruments, USA). The influence of space crowding (fractal dimension) was determined by adding 10 μg/mL of F-actin during the incubation reaction or with 10 ug/mL of polystyrene NPs of 50 nm diameter (Polyscience Inc., USA). The blanks consisted of freshly prepared F-actin buffer where ATP was replaced with ADP (no polymerization).

### 2.2 Exposure of mussels to polystyrene nanoparticles

Freshwater mussels *Elliptio complanata* were collected in a pristine lake under a provincial permit to limit any dangers of collecting mussels in local wild populations. For the exposure experiments, 4 mussels of similar size (6–8 cm shell length) were placed in 5 L glass containers (4 L exposure volume) and exposed to increasing concentrations (0.1, 0.5, 1 and 5 mg/L) of polystyrene uncoated nanosphere 50 nm diameter (Polyscience, USA) for 24 h at 15 °C under constant aeration. At the end of the exposure period, the mussels were allowed to stand in clean water for 24 h for depuration to allow the removal of loosely bound NPs and purging of the digestive gland contents. During the exposure period, dissolved oxygen, pH, conductivity, and temperature were monitored. The size distribution of these NPs were examined in MilliQ water and in aquarium water after 1 h using a dynamic light scatter instrument with an operating laser at 532 nm (Mobius Instrument, Wyatt Technologies, Santa Barbara, CA, USA). A concentration of 1 mg/L of 50 nm of NPs contained 2.575 x 10^10^ particles/L. These concentrations were selected to explore the potential effects of NPs and not expected to be found in the environment but could be reached during spillage situation of industrial sources of plastics. For example, the degradation of just one polystyrene coffee-cup cap led to the release of 150–200 million nanoparticles/mL of mean diameter of 200 nm after 58 days [26].

At the end of the experiment, mussels were dissected for the digestive gland mixed with 5 vol of 25 mM Hepes-NaOH, pH 7.4, containing 100 mM sucrose, 0.1 mM dithiothreitol and 1 μg/mL apoprotinin (proteases inhibitor) at 4 °C. The tissues were homogenized using a Teflon pestle tissue grinder (5 passes) and centrifuged at 10 000×*g* for 20 min at 2 °C. The supernatant was collected and stored at −85 °C until analysis. Preliminary experiments with NPs solutions revealed that they did not precipitate during centrifugation and freezing steps. Total proteins were determined in the homogenate and the supernatant using the Coomassie brilliant blue methodology [7]. Standard solutions of serum bovine albumin were used for calibration.

### 2.3 Detection of NPs in digestive gland and LDH activity

The levels of polystyrene NPs were determined in the supernatant using a molecular rotor probe as previously published [11]. The assay is based on the production of specific fluorescence emission peak (450 nm excitation, 620 nm emission) of the molecular rotor probe 9- (dicyanovinyl)-julolidine (DCVJ) in the presence of polystyrene NPs. This probe is normally used to determine viscosity changes at 520 nm with the same excitation [15]. The probe was dissolved at 10 mM in ethanol and diluted to 10 μM in MilliQ water at the day of analysis. A 20 μL volume of the supernatant was mixed with 180 μL of 10 μM DCVJ probe for 5 min. Fluorescence was taken at 450 nm excitation and 620 nm emission for polystyrene NPs. Standards of glycerol was used for calibration (0-20%). The activity of LDH was also determined in the S10 fraction of the digestive gland homogenate in the presence of 1 mM NADH and increasing amounts of pyruvate (0, 0.1, 0.2, 0.5, 0.75 and 1 mM) in 50 mM NaCl, 0.1 mM MgCl_2_, 10 μM DCVJ probe, 10 mM Hepes-NaOH, pH 7.4. The decrease in NADH (excitation 360 nm/emission 460 nm) and viscosity (excitation 450 nm/emission 520 nm) were measured each 30 s (in flash mode for quick readings) using a fluorescence microplate reader (Synergy-4, Biotek Instrument, USA). The data was expressed as decreased NADH relative fluorescence units/min/mg proteins. For viscosity, the data was expressed as relative fluorescence units (RFU)/mg proteins.

### 2.4 Data analysis

LDH reaction rate was determined by following the time-dependent decrease in NADH in free solution, crowded media by either F-actin or 50 nm NPs during the in vitro experiments or by the subcellular fraction of the digestive gland homogenate in mussels (N = 4 mussels per treatment) exposed to the NPs. The decrease pattern in NADH levels in time was analyzed using the Hurst exponent (rescaled range analysis) to derive the fractal dimension (Fractal analysis, Autosignal version 1.7 software package). The Hurst exponent is defined by R/S = k (t)^H^ where R the data range (Max-min) and S the standard deviation at a given time interval t and H is the Hurst exponent. In other terms, H is the slope of log transformation of the equation: log(R/S) = H log (t) + log C where C is a constant. The Hurst exponent is referred as the index of dependence and the tendency of a time series to take a pattern in time. A value of H = 0.5 represents white noise or pure random motion while values < 0.5 indicates a periodic change and values > 0.5 indicates long-term dependence of the signal over time. The fractal dimension (Df) was obtained simply by the following definition: H = 1/Df. The fractal kinetic constants were determined as previously described [17] where fK_M_= (V_Max_ S^(2−Df)^)/V–S where fK_M_ is the fractal Michaelis-Menten constant, V_Max_ is the maximal velocity, S the substrate concentration at a given reaction rate V and Df is the fractal dimension as explained above. The periodic changes in viscosity during the in vitro LDH reaction were analyzed by Fourier transformation (Harmonic analysis, Autosignal version 1.7) because of the oscillatory changes in viscosity over time. All experiments were repeated three times and the data analyzed using SYSTAT (version 13). Significance was set at p < 0.05.

## 3 Results and discussion

**Fig. 1:**
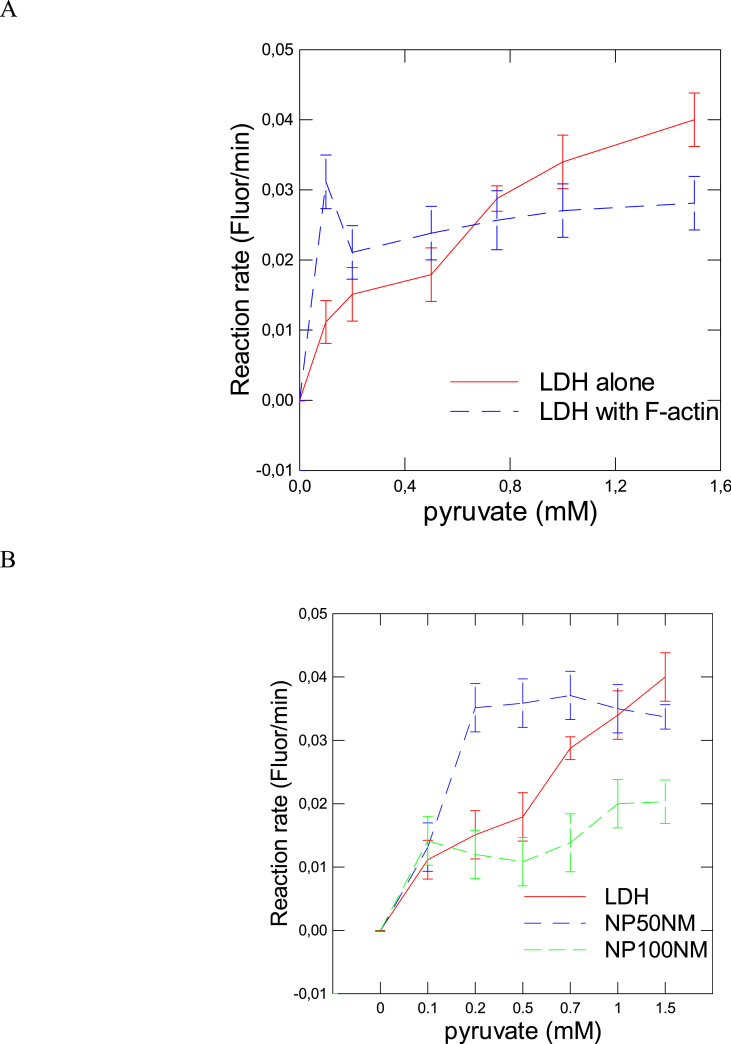
The influence of F-actin during the LDH reaction in vitro. The activity of LDH was determined with increasing concentrations of pyruvate in the presence of 1 mM NADH in the absence or presence of 10 μg/mL of F-actin (A) and with added 10 μg/mL polystyrene NPs of 50 and 100 nm diameter (B). The data represent the mean with the standard error.

The reaction rate catalyzed by LDH was examined in vitro in the absence or presence of F-actin, 50 and 100 nm NPs. As expected the reaction rate increases with increasing addition of pyruvate until saturation is reached at pyruvate concentration >1 mM (Fig. 1A). The addition of 10 μg/mL of F-actin in the reaction media displayed a bimodal response characteristic of fractal organisation of space. Indeed, at low pyruvate concentration, the reaction rate was increased by the presence of F-actin compared to the LDH reaction alone but the reaction decreased as the concentration of pyruvate increases. The same bimodal response was observed when 50 and 100 nm NPs were added to the reaction media (Fig. 1B). The reaction rates were higher at low substrate (pyruvate) concentration compared to LDH reaction alone followed with a steady decrease as the pyruvate concentration increases. However, the addition of 100 nm NPs did not produce a significant increase in the reaction rate at low substrate concentration but did reduce the rate at higher substrate concentration. This bimodal response curve is characteristic of enzyme reactions taking place in fractal space [3]. In a fractal space where dimensions are reduced, the substrates take shorter/quicker path to reach the enzyme at low concentrations compared in an unrestricted space where free diffusion dominates the reaction rates. However, these “paths” saturate more quickly than free solution and the reaction rates tend to decrease over time owing to crowding (traffic) effects. It appears that the presence of polystyrene NPs also leads to a fractal-like behavior in respect to the LDH reaction indicating that NPs could change the space environment of enzymes in a fractal manner. Polystyrene NPs are known to form heteroaggregates forming various paths similar to a percolation lattice [1] which could influence enzyme kinetics. This is consistent when enzymes interact at fractal surfaces such as enzymes are immobilized on solid surfaces for sensors [25]. Indeed, the addition of “surfaces” in the reaction media would tend to change the kinetics constants of enzymes in time. We also calculated the fK_M_ values based on the fractal dimension for the in vitro LDH reaction data (Table 1). The fractal dimension of the LDH reaction in controls was 1.176 and significantly decreased to 0.991 in the presence F-actin indicating a crowding effect on the reaction rates. The fractal dimension also decreased to 0.865 and 0.917 when 50 and 100 nm NPs were added in the reaction media respectively. Calculation of the fK_M_ values (see data analysis in Methods) revealed that the enzyme affinity significantly decreased in the presence of F-actin, 50 nm and 100 nm polystyrene NPs. This suggests that the addition of these crowding agents organized as fractal decrease the enzyme affinity to the substrates. In other words, the loss in enzyme affinity towards the substrates is a consequence of limited local substrate concentration in a crowded environment because the substrates would have to percolate through a “maze” of restricted (fractal) space to reach the enzymes. The cytoskeletal architecture, composed of F-actin and tubulin, is organized as a percolation lattice with clusters emerging in a fractal manner, i.e., the geometrical form of the clusters is expressed at different scales [3]. A consequence for this organization is that enzyme activity could be increased at low substrate concentrations but dampened as the concentration of the substrates is increased. This suggests that the cytoskeleton protein network are self-organizing and *in vivo* reactions initially runs faster than the same reaction in homogenous space [18]. However, crowding effects increase the substrate concentration locally in a restricted, fractal space and would tend to limit free diffusion process and decrease enzyme activity.

**Table 1:** Fractal dimension and fractal Km for LDH.

Conditions	Husrt exponent	Fractal dimension	Fractal Km (mM)
LDH alone	0.85 ± 0.003	1.176 ± 0.01	0.202
LDH with F-actin (10 μg/mL)	1.01 ± 0.015*	0.991 ± 0.02*	0.744
LDH with 50 nm NPs (1 μg/mL)	1.154 ± 0.013*	0.865 ± 0.02*	0.258
LDH with 100 nm NPs (1 μg/mL)	1.090 ± 0.02*	0.917 ± 0.02*	0.855

**Fig. 2:**
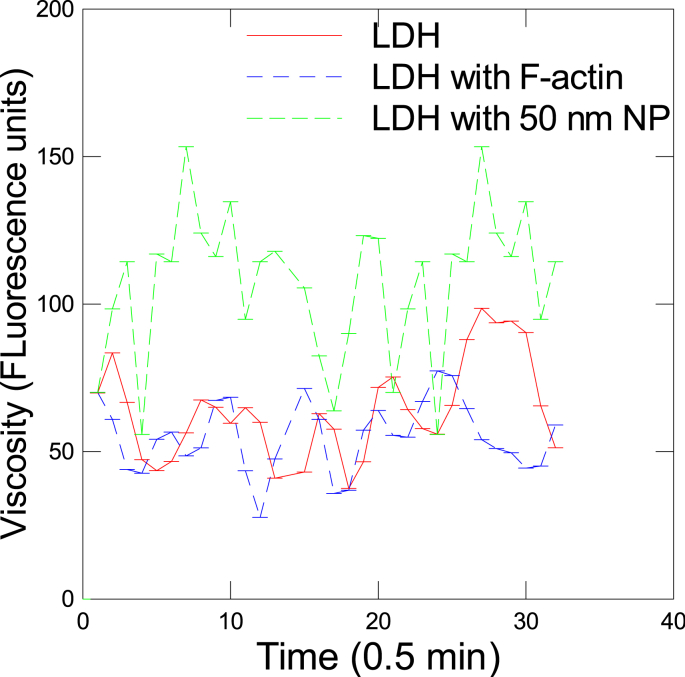
Change in viscosity by F-actin and NPs during the in vitro LDH reaction. Changes in viscosity during the LDH reaction in vitro were measured by a molecular rotor probe DCVJ. Fluorescence were measured each 0.5 min for 15 min at 450 nm excitation and 520 emission.

Changes in viscosity of the reaction media during the LDH reaction were also determined (Fig. 2). The data revealed that viscosity waves are produced during the oxidation of NADH with a period of 2.45 min in the control (unrestricted) reaction. In the presence of F-actin, the period was reduced to 2.05 min and some of the intensity signal was higher compared to LDH reaction only suggesting that crowding increased the frequency and somewhat the amplitudes in viscosity during the reaction. The addition of 50 nm NPs further increased the amplitudes and frequency in viscosity waves. A possible explanation for these results, the "movement" of LDH in solution is amplified when the space becomes restricted in a fractal manner. This could be a form of dynamic allostery where vibrations in the vicinity of the enzyme promotes reaction rates so-called rate-promoting vibrations of some enzymes such as LDH [10]. However, these vibrations occur at sub-microsecond scale, which were well below the range observed for viscosity waves. These vibrations could also be associated to the switching between the active and resting conformation of LDH [28]. These viscosity waves also suggests that the enzyme complexes exists in a relaxed (low viscosity) and tensed (high viscosity) states acting in a coherent or synchronized manner. The appearance of viscosity waves at higher frequencies for NPs suggests that the enzymes oscillates between high and low viscosity states more quickly and increased amplitudes in viscosity suggests that crowding by NPs leads to a more compressed state of the enzyme compared to F-actin which underlies the increased reaction rates at low substrate concentration.

**Fig. 3:**
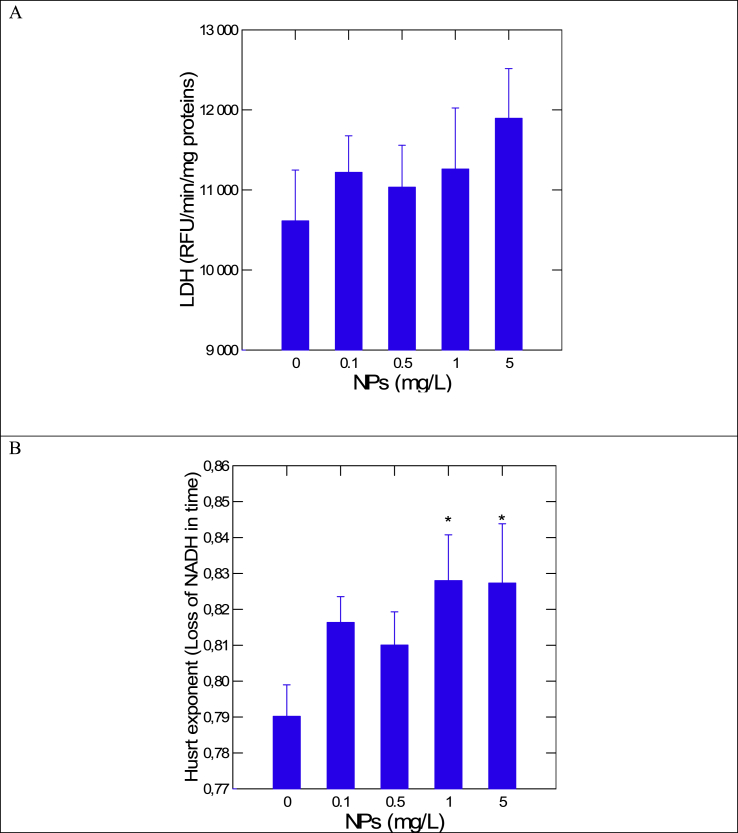
Rate change in LDH activity and fractal dimension in mussels exposed to polystyrene NPsIn mussels exposed to increasing concentration of 50 nm NPs, the activity in LDH (A) and Hurst exponent (B) were determined.

**Fig. 4:**
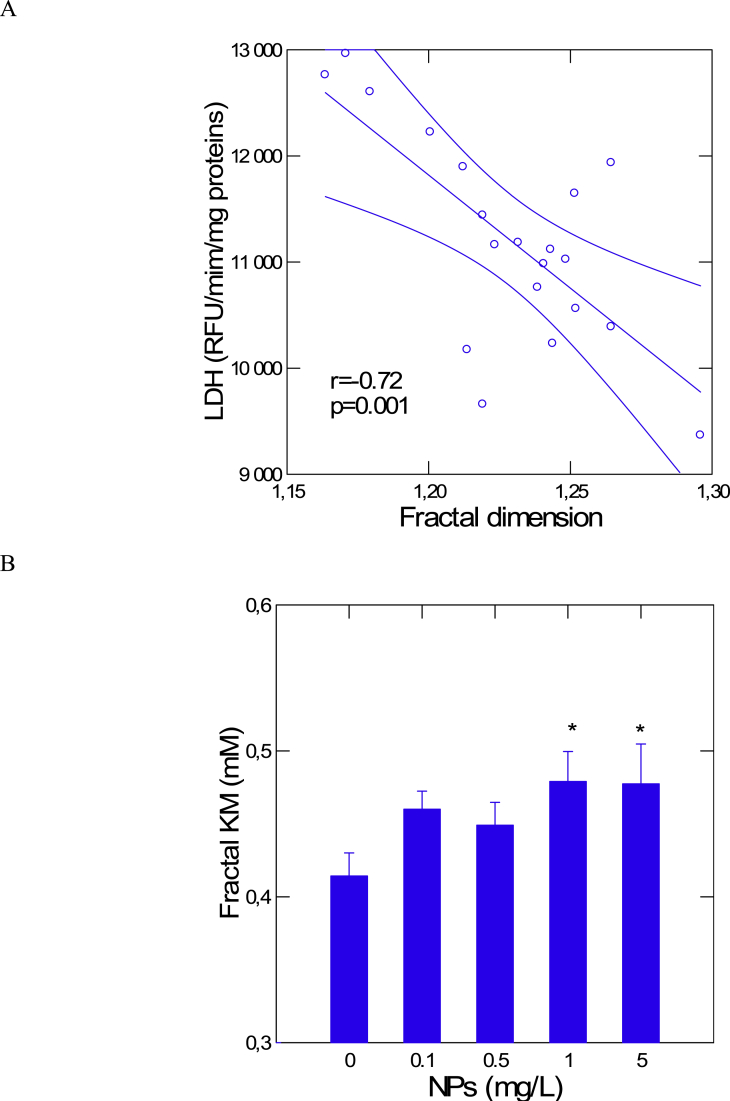
The influence of NPs on the fractal dimension and LDH substrate affinity. The fractal dimension was calculated from the Hurst exponent and plotted against LDH activity in the digestive gland of mussels exposed to NPs (A). The apparent fractal Km was calculated from the in vitro LDH data in Table 1 (B). The star symbol indicates significance at p < 0.05 level.

In the attempt to determine whether the above effects observed in vitro could arise in organisms exposed to NPs, *Elliptio complanata* mussels were exposed to increasing concentrations of polystyrene NPs (0.1–5 mg/L) in aquarium water for 24-48 h. Exposure of mussels to NPs led to increased concentration of NPs in the digestive gland with no changes in mussel conditions and lipid levels (Table 2). The activity of LDH and the Hurst exponent were determined in the subcellular fraction of the digestive gland in the presence of 0.1–1 mM pyruvate and 1 mM NADH (Fig. 3A and B). The specific LDH activity (at saturating pyruvate concentration of 1 mM) did not vary significantly although a marginal increase was observed at 5 mg/L NPs. The Hurst exponent was calculated from the time dependent decrease in NADH (Fig. 3B). The results revealed that the Hurst exponent was significantly increased at 1 and 5 mg/L indicating that the fractal dimension associated to LDH activity was reduced in the digestive gland. We found a strong negative relationship between the fractal dimension and LDH activity in freshwater mussels exposed to NPs (Fig. 4A). An analysis of covariance of LDH between the exposure concentration and the Hurst exponent revealed that only the covariate was significant suggesting that LDH activity was not influenced by other properties of NPs (i.e., chemically neutral). The relative fK_M_ was also calculated based on the in vitro LDH data (Table 1) and revealed a significant decrease in enzyme affinity (i.e., higher K_M_) for pyruvate in mussels exposed to 1 and 5 mg/L 50 nm NPs (Fig. 4B). This suggests that the NPs produce changes in the crowding of the intracellular (cytoskeleton) protein network, which decreased the affinity of LDH in the cytosol. The apparent decrease in affinity could result from the increased trafficking of the substrate across the protein maze in the S10 fraction. Thus, LDH activity could change by the spatial organisation of the cytosol which can be influenced by the sheer physical presence of nanoparticles. Changes in the biophysical properties of the cytoplasm by polystyrene NPs was also observed in hydra [5]. Indeed, exposure to 50 nm polystyrene NPs led to significant accumulation of NPs in tissues homogenate, formation of liquid crystals, viscosity and mobilization of polar lipids. These changes could produce changes in the spatial organisation of intracellular proteins and lipids as revealed in the present study. The formation of anisotropic liquid crystals was also observed in tissue extracts of the digestive gland spiked with polystyrene NPs and these were also observed in mussels collected downstream a municipal effluent which is a suspected source of (nano)plastics for the aquatic environment [12].

**Table 2:** Detection of polystyrene NPs in mussels.

Conditions (mg/L)	Condition factor (mussel weight/shell length)	Total lipids (mg/mg proteins)	Polystyrene NP (ng/mg proteins)
Controls	0.72 ± 0.04	2.0 ± 0.28	Nd
0.1	0.77 ± 0.05	2.26 ± 0.20	35 ± 11*
0.5	0.74 ± 0.07	2.0 ± 0.21	33 ± 13*
1	0.78 ± 0.03	2.3 ± 0.38	27 ± 6*
5	0.69 ± 0.06	2.5 ± 0.29	50 ± 8*

* significant from controls at p < 0.05.

In conclusion, polystryrene NPs was shown to reduce the fractal dimension of the LDH reaction in vitro and in the digestive gland of mussels exposed to NPs. This led to the characteristic increase in the reaction rates at low substrate concentration, which were followed by a deceleration of the reaction as the substrate concentration increased in the presence of crowding agents F-actin, 50 and 100 nm NPs. In the context that enzyme activities are determined at saturating amounts of substrate, the resulting rates could be reduced by reduced dimensions of space. The oscillatory changes in viscosity revealed that the LDH enzymes oscillated between 2 states of compressed (high viscosity) and relaxed conformation (low viscosity) and the presence of NPs increased the frequency and amplitudes of these oscillations at low substrate concentration where the rates were accelerated. In mussels exposed to polystryrene NPs, a decrease in the fractal dimension was also observed in the subcellular fraction of the digestive gland and explained most of the LDH activity in this fraction. A decrease in enzyme affinity was observed at the high concentrations of NPs (1 and 5 mg/L) suggesting that NPs could induce changes in the biophysical properties of the intracellular fraction in tissues and modulate enzyme activity.

## Declaration of competing interest

The authors declare not conflict of interest either of financial or personal in nature.
